# 3D Bioprinting amyloid plaque‐like deposits, a strategy to model Alzheimer's disease *in vitro*

**DOI:** 10.1002/alz70855_104715

**Published:** 2025-12-24

**Authors:** Stefano Sorrentino, Declan J Brennan, Stefan Wendt, Christopher Lee, Wenji Cai, Xiujuan Wu, Haakon B. Nygaard

**Affiliations:** ^1^ University of British Columbia, Vancouver, BC, Canada

## Abstract

**Background:**

Amyloid β (Aβ) aggregation is a key neuropathological hallmark of Alzheimer's disease (AD) and is believed to trigger the pathological cascade leading to neurodegeneration. However, despite decades of research, the mechanisms underlying amyloidogenesis remain unclear, partly due to the lack of representative models that accurately recapitulate human pathology. Given that Aβ aggregates form in the extracellular space through the interaction with the extracellular matrix (ECM), conventional 2D cultures are inadequate for studying this process. Conversely, *in vivo* models provided insights but failed to fully replicate human Aβ aggregation dynamics, neurotoxicity, and disease progression. Recent advances in 3D bioprinting now allow the creation of physiologically relevant human brain models by integrating human induced pluripotent stem cells (hiPSCs) with ECM‐like biomaterials (bioinks).

**Method:**

Here, we developed a 3D bioprinted human brain model for long‐term culture of iPSC‐derived familial AD (fAD) cortical neurons, astrocytes, and microglia in a multilayer wood‐pile structure that mimics the cytoarchitecture of the human cortex. To model Aβ plaque formation, we incorporated synthetic fibrillar Aβ42 (fAβ42) into the bioink, leveraging AβV717I mutant neurons as a substrate while using fAβ42 as a seed to promote aggregation.

**Result:**

After three weeks of culture, we observed a statistically significant decrease in endogenous Aβ40, Aβ42, and the Aβ40/Aβ42 ratio in the conditioned medium, suggesting increased Aβ aggregation. Immunostaining confirmed increased accumulation of mOC87 and 4G8‐positive Aβ deposits within the 3D‐printed constructs.

**Conclusion:**

Our findings demonstrate the feasibility of mimicking amyloidogenesis in a 3D human‐derived brain model, overcoming limitations of traditional *in vitro* models. This approach provides a human‐relevant platform to study Aβ nucleation and plaque formation in real‐time, offering a novel tool for AD research and drug discovery without relying on animal models.

